# Monotone Data Visualization Using Rational Trigonometric Spline Interpolation

**DOI:** 10.1155/2014/602453

**Published:** 2014-04-03

**Authors:** Farheen Ibraheem, Maria Hussain, Malik Zawwar Hussain

**Affiliations:** ^1^National University of Computer and Emerging Sciences, Lahore, Pakistan; ^2^Department of Mathematics, Lahore College for Women University, Lahore 54600, Pakistan; ^3^Department of Mathematics, University of the Punjab, Lahore 54590, Pakistan

## Abstract

Rational cubic and bicubic trigonometric schemes are developed to conserve monotonicity of curve and surface data, respectively. The rational cubic function has four parameters in each subinterval, while the rational bicubic partially blended function has eight parameters in each rectangular patch. The monotonicity of curve and surface data is retained by developing constraints on some of these parameters in description of rational cubic and bicubic trigonometric functions. The remaining parameters are kept free to modify the shape of curve and surface if required. The developed algorithm is verified mathematically and demonstrated graphically.

## 1. Introduction

The technique or algorithm employed in creating images, diagrams, or animations for imparting a piece of information is termed as visualization. It has a key role to play in different fields like science, engineering, education, and medicine as it can aid experts in identifying and interpreting different patterns and artifacts in their data and provide a three-dimensional display of data for the solution of a wide range of problems.

The methods used to obtain visual representations from abstract data have been in practice for a long time. However, physical quantities often emanate distinctive features (such as positivity, convexity, and monotonicity) and it becomes imperative that the visual model must contain the shape feature to fathom the physical phenomenon, the scientific experiment, and the idea of the designer. Spline interpolating functions play elemental role in visualizing shaped data. This paper specifically addresses the problem of visualizing monotone curve and surface data.

Monotonicity is an indispensable characteristic of data stemming from many physical and scientific experiments. The relationship between the partial pressure of oxygen and percentage dissociation of hemoglobin, consumption function in economics, concentration of atrazine and nitrate in shallow ground waters, and approximation of couples and quasi couples are few phenomena which exhibit monotone trend.

Efforts have been put in by many researchers and a variety of approaches has been proposed to solve this eminent issue [1–17]. Cripps and Hussain [3] visualized the 2D monotone data by Bernstein-Bézier rational cubic function. The authors in [3] converted the Bernstein-Bézier rational cubic function to *C* ^1^ cubic Hermite by applying the *C* ^1^ continuity conditions at the end points of interval. The lower bounds of weights functions were determined to visualize monotone data as monotone curve. Hussain and Sarfraz [8] have conserved monotonicity of curve data by rational cubic function with four shape parameters, two of which were set free and two were shape parameters. Data dependent constraints on shape parameters were developed which assure the monotonicity but one shape parameter is dependent on the other which makes it economically very expensive. Rational cubic function with two shape parameters suggested by Sarfraz [13] sustained monotonicity of curves but lacked the liberty to amend the curve which makes it inappropriate for interactive design. Piecewise rational cubic function was used by M. Z. Hussain and M. Hussain [7] to visualize 2D monotone data by developing constraints on the free parameters in the specification of rational cubic function. The authors also extended rational cubic function to rational bicubic partially blended function. Simple constraints were derived on the free parameters in the description of rational bicubic partially blended patches to visualize the 3D monotone data. Three kinds of monotonicity preservation of systems of bivariate functions on triangle were defined and studied by Floater and Peña [5]. Sarfraz et al. [12] developed constraints in the specification of a bicubic function to visualize the shape of 3D monotone data.

This paper is a noteworthy addition in the field of shape preservation when the data under consideration admits monotone trend. The suggested algorithm offers numerous advantages over the prevailing ones. Orthogonality of sine and cosine function compels much smoother visual results as compared to algebraic spline. Derivative of the trigonometric spline is much lower than that of algebraic spline. Moreover, trigonometric splines play an instrumental role in robotic manipulator path planning.

The remainder of the paper is structured as follows.  Section 2 is devoted to reviewing the rational trigonometric cubic function developed in [11]. In Section 3, rational trigonometric cubic function is extended to rational trigonometric bicubic function.  Section 4 aims to develop monotonicity preserving constraints for 2D data.  Section 5 submits a solution to shape preservation of 3D monotone data. In Section 6, numerical examples have been demonstrated.  Section 7 draws the conclusion and significance of this research.

## 2. Rational Trigonometric Cubic Function

In this section, rational trigonometric cubic function [11] is reviewed.

Let {(*x*_*i*_, *f*_*i*_), *i* = 0,1, 2,…, *n*} be the given set of data points defined over the interval [*a*, *b*], where *a* = *x*_0_ < *x*_1_ < *x*_2_ < ⋯<*x*_*n*_ = *b*. Piecewise rational trigonometric cubic function is defined over each subinterval *I*_*i*_ = [*x*_*i*_, *x*_*i*+1_] as

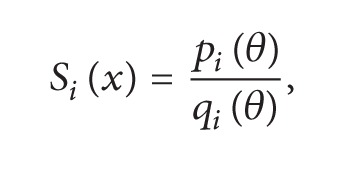
(1)

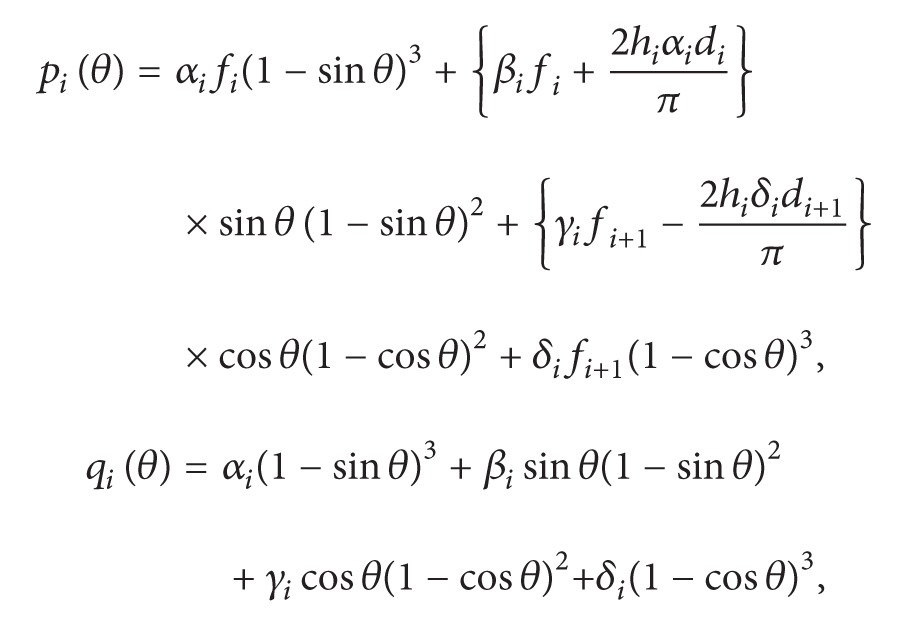
(2)
where *θ* = (*π*/2)((*x* − *x*_*i*_)/*h*_*i*_), *h*_*i*_ = *x*_*i*+1_ − *x*_*i*_.

The rational trigonometric cubic function (1) is *C* ^1^; that is, it satisfies the following properties:

(3) $$\begin{array}{c} S\left(x_{i}\right)=f_{i},\;\;S\left(x_{i+1}\right)=f_{i+1},\\ S^{\prime }\left(x_{i}\right)=d_{i},\;\;S^{\prime }\left(x_{i+1}\right)=d_{i+1}. \end{array}$$

Here *d*_*i*_ and *d*_*i*+1_ are derivatives at the end points of the interval *I*_*i*_ = [*x*_*i*_, *x*_*i*+1_]. The parameters *α*_*i*_ and *δ*_*i*_ are real numbers used to modify the shape of the curve.

## 3. Rational Trigonometric Bicubic Partially Blended Function

Let {(*x*_*i*_, *y*_*j*_, *F*_*i*,*j*_), *i* = 0, 1, 2,…, *n* − 1; *j* = 0, 1, 2,…, *m* − 1} be the 3D regular data set defined over the rectangular mesh *I* = [*a*, *b*] × [*c*, *d*], let *p* : *a* = *x*_0_ < *x*_1_ < ⋯<*x*_*m*_ = *b* be a partition of [*a*, *b*], and let *q* : *a* = *y*_0_ < *y*_1_ < ⋯<*y*_*n*_ be a partition of [*c*, *d*]. Rational trigonometric bicubic function which is an extension of rational trigonometric cubic function (1) is defined over each rectangular patch [*x*_*i*_, *x*_*i*+1_] × [*y*_*j*_, *y*_*j*+1_], where *i* = 0, 1, 2,…, *n* − 1; *j* = 0, 1, 2,…, *m* − 1, as

(4) $$\begin{array}{c} S\left(x,y\right)=-AFB^{T}, \end{array}$$

where

(5) $$\begin{array}{c} F=\left(\begin{array}{ccc} 0 & S\left(x,y_{j}\right) & S\left(x,y_{j+1}\right)\\ S\left(x_{i},y\right) & S\left(x_{i},y_{j}\right) & S\left(x_{i},y_{j+1}\right)\\ S\left(x_{i+1},y\right) & S\left(x_{i+1},y_{j}\right) & S\left(x_{i+1},y_{j+1}\right) \end{array}\right),\\ A=\left[\begin{array}{ccc} -1 & a_{0}\left(\theta \right) & a_{1}\left(\theta \right) \end{array}\right],\;\;B=\left[\begin{array}{ccc} -1 & b_{0}\left(\theta \right) & b_{1}\left(\theta \right) \end{array}\right],\\ a_{0}=\cos^{2}\theta ,\;\;a_{1}=\sin^{2}\theta ,\\ b_{0}=\cos^{2}\varphi ,\;\;b_{1}=\sin^{2}\varphi . \end{array}$$

*S*(*x*, *y*_*j*_), *S*(*x*, *y*_*j*+1_), *S*(*x*_*i*_, *y*), and *S*(*x*_*i*+1_, *y*) are rational trigonometric bicubic functions defined on the boundary of rectangular patch [*x*_*i*_, *x*_*i*+1_] × [*y*_*j*_, *y*_*j*+1_] as

(6) S(x,yj)=(A0(1−sin⁡θ)3+A1sin⁡θ(1−sin⁡θ)2 +A2cos⁡⁡θ(1−cos⁡⁡θ)2+A3(1−cos⁡⁡θ)3)×(q1(θ))−1,

where

(7) A0=αi,jFi,j,  A1=βi,jFi,j+2αi,jhiFi,jxπ,A2=γi,jFi+1,j−2δi,jhiFi+1,jxπ,  A3=δi,jFi+1,j,q1(θ)=αi,j(1−sin⁡θ)3+βi,jsin⁡θ(1−sin⁡θ)2+γi,jcos⁡⁡θ⁡(1−cos⁡⁡θ)2+δi,j(1−cos⁡⁡θ)3,

(8) S(x,yj+1)=(B0(1−sin⁡θ)3+B1sin⁡θ(1−sin⁡θ)2 +B2cos⁡⁡θ(1−cos⁡⁡θ)2+B3(1−cos⁡⁡θ)3)×(q2(θ))−1,

where

(9) B0=αi,j+1Fi,j+1,  B1=βi,j+1Fi,j+1+2αi,j+1hiFi,j+1xπ,B2=γi,j+1Fi+1,j+1−2δi,j+1hiFi+1,j+1xπ,  B3=δi,j+1Fi+1,j+1,q2(θ)=αi,j+1(1−sin⁡θ)3+βi,j+1sin⁡θ(1−sin⁡θ)2+γi,j+1cos⁡⁡θ(1−cos⁡⁡θ)2+δi,j+1(1−cos⁡⁡θ)3,

(10) S(xi,y)=(C0(1−sin⁡φ)3+C1sin⁡φ(1−sin⁡φ)2 +C2cos⁡⁡φ(1−cos⁡⁡φ)2+C3(1−cos⁡⁡φ)3)×(q3(φ))−1,

where

(11) C0=α^i,jFi,j,  C1=β^i,jFi,j+2α^i,jhjFi,jyπ,C2=γ^i,jFi,j+1−2δ^i,jhjFi,j+1yπ,  C3=δ^i,jFi,j+1,q3(φ)=α^i,j(1−sin⁡φ)3+β^i,jsin⁡φ(1−sin⁡φ)2+γ^i,jcos⁡⁡φ(1−cos⁡⁡φ)2+δ^i,j(1−cos⁡⁡φ)3,

(12) S(xi+1,y)=(D0(1−sin⁡φ)3+D1sin⁡φ(1−sin⁡φ)2 +D2cos⁡⁡φ(1−cos⁡⁡φ)2+D3(1−cos⁡⁡φ)3)×(q4(φ))−1,

where

(13) D0=α^i+1,jFi+1,j,  D1=β^i+1,jFi,j+1+2α^i+1,jhjFi+1,jyπ,D2=γ^i+1,jFi+1,j+1−2δ^i+1,jhjFi+1,j+1yπ,D3=δ^i+1,jFi+1,j+1,q4(φ)=α^i+1,j(1−sin⁡φ)3+β^i+1,jsin⁡φ(1−sin⁡φ)2+γ^i+1,jcos⁡⁡φ⁡(1−cos⁡⁡φ)2+δ^i+1,j(1−cos⁡⁡φ)3.

## 4. Monotone Curve Interpolation

Monotonicity is a crucial shape property of data and it emanates from many physical phenomenon, engineering problems, scientific applications, and so forth, for instance, dose response curve in biochemistry and pharmacology, approximation of couples and quasi couples in statistics, empirical option pricing model in finance, consumption function in economics, and so forth. Therefore, it is customary that the resulting interpolating curve must retain the monotone shape of data.

In this section, constraints on shape parameters in the description of rational trigonometric cubic function (1) have been developed to preserve 2D monotone data.

Let {(*x*_*i*_, *f*_*i*_), *i* = 0,1, 2,…, *n*} be the monotone data defined over the interval [*a*, *b*]; that is,

(14) $$\begin{array}{c} f_{i}<f_{i+1},\;\Delta_{i}=\frac{f_{i+1}-f_{i}}{h_{i}}>0,\;i=0,1,2,\ldots ,n-1,\\ d_{i}>0,\;i=0,1,2,\ldots ,n. \end{array}$$

The curve will be monotone if the rational trigonometric cubic function (1) satisfies the condition

(15) $$\begin{array}{c} S_{i}^{\prime }\left(x\right),\;\forall x\in \left[x_{i},x_{i+1}\right],\;\;i=0,1,2,\ldots ,n-1. \end{array}$$

Now, we have

(16) Si′(x) =π2hi(qi(θ))2  ×{(1−sin⁡θ)4sin⁡θcos⁡⁡θB0     +cos⁡2⁡θ(1−cos⁡⁡θ)2(1−sin⁡θ)2B1     +cos⁡⁡θ(1−cos⁡⁡θ)3(1−sin⁡θ)2B2     +cos⁡⁡θ(1−sin⁡θ)5B3+sin⁡θcos⁡2⁡θ(1−cos⁡⁡θ)2     ×(1−sin⁡θ)B4+sin⁡θcos⁡⁡θ(1−sin⁡θ)(1−cos⁡⁡θ)3B5     +sin⁡θ(1−cos⁡⁡θ)2(1−sin⁡θ)3B6     +sin2⁡θ(1−sinθ)2(1−cos⁡⁡θ)2B7     +sin⁡θcos⁡⁡θ(1−cos⁡⁡θ)4B8+sin⁡θ(1−cos⁡⁡θ)5B9     +sin⁡θcos⁡⁡θ(1−cos⁡θ)(1−sin⁡θ)3B10     +sin2⁡θcos⁡⁡θ(1−cos⁡⁡θ)(1−sin⁡θ)2B11},

where

(17) B0=2hidiαi2π,B1=(3αiγi−βiδi)Δi+2diαiγiπ−(3αi−βi)2δidi+1π,B2=(βi−3αi)Δi−2diαiπ,  B3=2hiαidiπ,B4=2βiγiΔi−4δiβidi+1π−4αiγidiπ,B5=βiΔi−2αidiπ,B6=(3δi−γi)Δi+2αidi+1π,B7=βi(γi−3δi)Δi+2αiγidiπ+(βi−3αi)2δidi+1π,B8=2hiδidi+1π,  B9=2hiδidi+1π,B10=γiΔi−2δidi+1π,B11=βiγiΔi−2βiδidi+1π−2αiγidiπ.

The denominator in (16) is a squared quantity, thus, positive. Hence, monotonicity of rational trigonometric cubic spline depends upon the positivity of numerator which can be attained if the coefficients *B*_*i*_, *i* = 0, 1, 2,…, 11 of the trigonometric basis functions are all positive. This yields the following result:

(18) $$\begin{array}{c} \beta_{i}>\frac{2\alpha_{i}d_{i}}{\pi \Delta_{i}},\;\;\gamma_{i}>\frac{2\delta_{i}d_{i+1}}{\pi \Delta_{i}}. \end{array}$$

The above discussion can be summarized as follows.

**Theorem 1**

The *C* ^1^ piecewise trigonometric rational cubic function (1) preserves the monotonicity of monotone data if in each subinterval *I*_*i*_ = [*x*_*i*_, *x*_*i*+1_], the parameters *β*_*i*_ and *γ*_*i*_ satisfy the following sufficient conditions:

(19) $$\begin{array}{c} \beta_{i}>\frac{2\alpha_{i}d_{i}}{\pi \Delta_{i}},\;\;\gamma_{i}>\frac{2\delta_{i}d_{i+1}}{\pi \Delta_{i}}. \end{array}$$

The above constraints can be rearranged as

(20) $$\begin{array}{c} \beta_{i}=u_{i}+\max\left\{0,\frac{2\alpha_{i}d_{i}}{\pi \Delta_{i}}\right\},\;u_{i}>0;\\ \gamma_{i}=v_{i}+\max\left\{0,\frac{2\delta_{i}d_{i+1}}{\pi \Delta_{i}}\right\},\;v_{i}>0. \end{array}$$

**Algorithm 2**

*Step 1.* Take a monotone data set {(*x*_*i*_, *f*_*i*_) : *i* = 0,1, 2,…, *n*}.

*Step 2.* Use the Arithmetic Mean Method [11] to estimate the derivatives *d*_*i*_'s at knots *x*_*i*_'s (note: Step 2 is only applicable if data is not provided with derivatives).

*Step 3.* Compute the values of parameters *β*_*i*_'s and *γ*_*i*_'s using Theorem 1.

*Step 4.* Substitute the values of variables from Steps 1–3 in rational trigonometric cubic function (1) to visualize monotone curve through monotone data.

## 5. Monotone Surface Interpolation

Let {(*x*_*i*_, *y*_*j*_, *F*_*i*,*j*_), *i* = 0, 1, 2,…, *n* − 1; *j* = 0, 1, 2,…, *m* − 1} be the monotone data set defined over the rectangular mesh *I* = [*x*_*i*_, *x*_*i*+1_] × [*y*_*j*_, *y*_*j*+1_] such that

(21) $$\begin{array}{c} F_{i,j}<F_{i+1,j},\;\;F_{i,j}<F_{i,j+1},\\ F_{i,j}^{x}>0,\;\;F_{i,j}^{y}>0,\\ \Delta_{i,j}>0,\;\;{\hat{\Delta }}_{i,j}>0. \end{array}$$

Now, surface patch (4) is monotone if the boundary curves defined in (6)–(12) are monotone.

Now, *S*(*x*, *y*_*j*_) is monotone if *S*_*i*_′(*x*, *y*_*j*_) > 0, where

(22) Si′(x,yj) =π2hi(q1(θ))2  ×{(1−sin⁡θ)4sin⁡θcos⁡⁡θR0     +cos⁡2⁡θ(1−cos⁡⁡θ)2(1−sin⁡θ)2R1     +cos⁡⁡θ(1−cos⁡⁡θ)3(1−sin⁡θ)2R2     +cos⁡⁡θ(1−sin⁡θ)5R3     +sinθcos⁡2⁡θ(1−cos⁡⁡θ)2(1−sin⁡θ)R4     +sin⁡θcos⁡⁡θ(1−sin⁡θ)(1−cos⁡⁡θ)3R5     +sin⁡θ(1−cos⁡⁡θ)2(1−sin⁡θ)3R6     +sin2⁡θ(1−sin⁡θ)2(1−cos⁡⁡θ)2R7     +sin⁡θcos⁡⁡θ(1−cos⁡⁡θ)4R8+sin⁡θ(1−cos⁡⁡θ)5R9     +sin⁡θcos⁡⁡θ(1−cos⁡⁡θ)(1−sin⁡θ)3R10     +sin2⁡θcos⁡⁡θ(1−cos⁡θ)(1−sin⁡θ)2R11},

with

(23) R0=2hiFi,jxαi,j2π,R1=(3αi,jγi,j−βi,jδi,j)Δi,j+2Fi,jxαi,jγi,jπ−(3αi,j−βi,j)2δiFi+1,jxπ,R2=(βi,j−3αi,j)Δi,j−2Fi,jxαi,jπ,  R3=2hiαi,jFi,jxπ,R4=2βi,jγi,jΔi,j−4δi,jβi,jFi+1,jxπ−4αi,jγi,jFi,jxπ,R5=βi,jΔi,j−2αi,jFi,jxπ,R6=(3δi,j−γi,j)Δi,j+2αi,jFi+1,jxπ,R7=βi,j(γi,j−3δi,j)Δi,j+2αi,jγi,jFi,jxπ+(βi,j−3αi,j)2δi,jFi+1,jxπ,R8=2hiδi,jFi+1,jxπ,  R9=2hiδi,jFi+1,jxπ,R10=γi,jΔi,j−2δi,jFi+1,jxπ,R11=βi,jγi,jΔi,j−2βi,jδi,jFi+1,jxπ−2αi,jγi,jFi,jxπ.

Now the positivity of *S*_*i*_′(*x*, *y*_*j*_) entirely depends on *R*_*i*_, *i* = 0,1, 2 …, 11. The denominator in (22) is always positive. Since the parameter *θ* lies in first quadrant therefore the trigonometric basis functions will be positive also. This yields the following constraints on the free parameters:

(24) $$\begin{array}{c} \beta_{i,j}>\frac{2\alpha_{i,j}F_{i,j}^{x}}{\pi \Delta_{i,j}},\;\;\gamma_{i,j}>\frac{2\delta_{i,j}F_{i+1,j}^{x}}{\pi \Delta_{i,j}}. \end{array}$$

*S*(*x*, *y*_*j*+1_) is monotone if

(25) $$\begin{array}{c} S_{i}^{\prime }\left(x,y_{j+1}\right)>0, \end{array}$$

where

(26) Si′(x,yj+1) =π2hi(q2(θ))2  ×{(1−sin⁡θ)4sinθcos⁡⁡θT0     +cos⁡2⁡θ(1−cos⁡⁡θ)2(1−sin⁡θ)2T1     +cos⁡⁡θ(1−cos⁡⁡θ)3(1−sin⁡θ)2T2     +cos⁡⁡θ(1−sin⁡θ)5T3     +sin⁡θcos⁡2⁡θ(1−cos⁡⁡θ)2(1−sin⁡θ)T4     +sin⁡θcos⁡⁡θ(1−sin⁡θ)(1cos⁡⁡θ)3T5     +sin⁡θ(1−cos⁡⁡θ)2(1−sin⁡θ)3T6     +sin2⁡θ(1−sin⁡θ)2(1−cos⁡⁡θ)2T7     +sin⁡θcos⁡⁡θ(1−cos⁡⁡θ)4T8+sin⁡θ(1cos⁡⁡θ)5T9     +sin⁡θcos⁡⁡θ(1−cos⁡⁡θ)(1−sin⁡θ)3T10     +sin2⁡θcos⁡⁡θ(1−cos⁡⁡θ)⁡(1−sin⁡θ)2T11},

with

(27) T0=2hiFi,j+1xαi,j+12π,T1=(3αi,j+1γi,j+1−βi,j+1δi,j+1)Δi,j+1+2Fi,j+1xαi,j+1γi,j+1π−(3αi,j+1−βi,j+1)2δi,j+1Fi+1,j+1xπ,T2=(βi,j+1−3αi,j+1)Δi,j+1−2Fi,j+1xαi,j+1π,T3=2hiαi,j+1Fi,j+1xπ,T4=2βi,j+1γi,j+1Δi,j+1−4δi,j+1βi,j+1Fi+1,j+1xπ−4αi,j+1γi,j+1Fi,j+1xπ,T5=βi,j+1Δi,j+1−2αi,j+1Fi,j+1xπ,T6=(3δi,j+1−γi,j+1)Δi,j+1+2αi,j+1Fi+1,j+1xπ,T7=βi,j+1(γi,j+1−3δi,j+1)Δi,j+1+2αi,j+1γi,j+1Fi,j+1xπ+(βi,j+1−3αi,j+1)2δi,j+1Fi+1,j+1xπ,T8=2hiδi,j+1Fi,j+1xπ,  T9=2hiδi,j+1Fi+1,j+1xπ,T10=γi,j+1Δi,j+1−2δi,j+1Fi+1,j+1xπ,T11=βi,j+1γi,j+1Δi,j+1−2βi,j+1δi,j+1Fi+1,j+1xπ−2αi,j+1γi,j+1Fi,j+1xπ.

The denominator in (26) is always positive. Moreover, the trigonometric basis functions are also positive for 0 ≤ *θ* ≤ *π*/2. It follows that the positivity of *S*_*i*_′(*x*, *y*_*j*+1_) entirely depends upon *T*_*i*_,   *i* = 0,1, 2 …, 11. This yields the following constraints on the free parameters:

(28) $$\begin{array}{c} \beta_{i,j+1}>\frac{2\alpha_{i,j+1}F_{i,j+1}^{x}}{\pi \Delta_{i,j+1}},\;\;\gamma_{i,j+1}>\frac{2\delta_{i,j+1}F_{i+1,j+1}^{x}}{\pi \Delta_{i,j+1}}. \end{array}$$

*S*(*x*_*i*_, *y*) is monotone if *S*_*i*_′(*x*_*i*_, *y*) > 0. We have

(29) Si′(xi,y) =π2hi(q3(φ))2  ×{(1−sin⁡φ)4sin⁡φcos⁡⁡φU0    +cos⁡2⁡φ(1−cos⁡⁡φ)2(1−sin⁡φ)2U1    +cos⁡⁡φ(1−cos⁡⁡φ)3(1−sin⁡φ)2U2    +cos⁡⁡φ(1−sin⁡φ)5U3    +sin⁡φcos⁡2⁡φ(1−cos⁡⁡φ)2(1−sin⁡φ)U4    +sin⁡φcos⁡⁡φ(1−sin⁡φ)(1cos⁡⁡φ)3U5    +sin⁡φ(1−cos⁡⁡φ)2(1−sin⁡φ)3U6    +sin2⁡φ(1−sin⁡φ)2(1−cos⁡⁡φ)2U7    +sin⁡φcos⁡⁡φ(1−cos⁡⁡φ)4U8+sin⁡φ(1cos⁡⁡φ)5U9    +sin⁡φcos⁡⁡φ(1−cos⁡⁡φ)(1−sin⁡φ)3U10    +sin2⁡φcos⁡⁡φ(1−cos⁡⁡φ)(1−sin⁡φ)2U11},

where

(30) U0=2hjFi,jyα^i,j2π,U1=(3α^i,jγ^i,j−β^i,jδ^i,j)Δ^i,j+2Fi,jyα^i,jγ^i,jπ−(3α^i,j−β^i,j)2δ^i,jFi,j+1yπ,U2=(β^i,j−3α^i,j)Δ^i,j−2Fi,jyα^i,jπ,U3=2hjα^i,jFi,jyπ,U4=2β^i,jγ^i,jΔ^i,j−4δ^i,jβ^i,jFi,j+1yπ−4α^i,jγ^i,jFi,jyπ,U5=β^i,jΔ^i,j−2α^i,jFi,jyπ,U6=(3δ^i,j−γ^i,j)Δ^i,j+2α^i,jFi,j+1yπ,U7=β^i,j(γ^i,j−3δ^i,j)Δ^i,j+2α^i,jγ^i,jFi,jyπ+(β^i,j−3α^i,j)2δ^i,jFi,j+1yπ,U8=2hjδ^i,jFi,j+1yπ,U9=2hjδ^i,jFi,j+1yπ,  U10=γ^i,jΔ^i,j−2δ^i,jFi,j+1yπ,U11=β^i,jγ^i,jΔ^i,j−2β^i,jδ^i,jFi,j+1yπ−2α^i,jγ^i,jFi,jyπ.

Since the denomoinator of (29) is always positive and trigonometric basis functions are positive for so the positivity of 0 ≤ *φ* ≤ *π*/2. It follows that the positivity of *S*_*i*_′(*x*_*i*+1_, *y*) entirely depends upon *U*_*i*_,   *i* = 0,1, 2 …, 11. This yields the following constraints on the free parameters:

(31) $$\begin{array}{c} {\hat{\beta }}_{i,j}>\frac{2{\hat{\alpha }}_{i,j}F_{i,j}^{y}}{\pi {\hat{\Delta }}_{i,j}},\;\;{\hat{\gamma }}_{i,j}>\frac{2{\hat{\delta }}_{i,j}F_{i,j+1}^{y}}{\pi {\hat{\Delta }}_{i,j}}. \end{array}$$

*S*(*x*_*i*+1_, *y*) is monotone if *S*_*i*_′(*x*_*i*+1_, *y*) > 0. We have

(32) Si′(xi+1,y) =π2hi(q4(φ))2  ×{(1−sin⁡φ)4sin⁡φcos⁡⁡φV0    +cos⁡2⁡φ(1−cos⁡⁡φ)2(1−sin⁡φ)2V1    +cos⁡⁡φ(1−cos⁡⁡φ)3(1−sin⁡φ)2V2    +cos⁡⁡φ(1−sin⁡φ)5V3    +sin⁡φcos⁡2⁡φ(1−cos⁡⁡φ)2(1−sin⁡φ)V4    +sin⁡φcos⁡⁡φ(1−sin⁡φ)(1cos⁡⁡φ)3V5    +sin⁡φ(1−cos⁡⁡φ)2(1−sin⁡φ)3V6    +sin2⁡φ(1−sinφ)2(1−cos⁡⁡φ)2V7    +sin⁡φcos⁡⁡φ(1−cos⁡⁡φ)4V8+sin⁡φ⁡(1cos⁡⁡φ)5V9    +sin⁡φcos⁡⁡φ(1−cos⁡φ)⁡(1−sin⁡φ)3V10    +sin2φcos⁡φ(1−cos⁡φ)(1−sin⁡φ)2V11},

where

(33) V0=2hjFi+1,jyα^i+1,j2π,V1=(3α^i+1,jγ^i,j−β^i+1,jδ^i+1,j)Δ^i+1,j+2Fi+1,jyα^i+1,jγ^i+1,jπ−(3α^i+1,j−β^i+1,j)2δ^i+1,jFi+1,j+1yπ,V3=2hjα^i+1,jFi+1,jyπ,V4=2β^i+1,jγ^i+1,jΔ^i+1,j−4δ^i+1,jβ^i+1,jFi+1,j+1yπ−4α^i+1,jγ^i+1,jFi+1,jyπ,V5=β^i+1,jΔ^i+1,j−2α^i+1,jFi+1,jxπ,V6=(3δ^i+1,j−γ^i+1,j)Δ^i,j+2α^i+1,jFi+1,j+1xπ,V7=β^i+1,j(γ^i+1,j−3δ^i+1,j)Δ^i+1,j+2α^i+1,jγ^i+1,jFi,jyπ+(β^i+1,j−3α^i+1,j)2δ^i,jFi+1,j+1yπ,V8=2hjδ^i+1,jFi+1,j+1yπ,  V9=2hjδ^i+1,jFi+1,j+1xπ,V10=γ^i+1,jΔ^i+1,j−2δ^i+1,jFi+1,j+1yπ,V11=β^i+1,jγ^i+1,jΔ^i+1,j−2β^i+1,jδ^i+1,jFi+1,j+1yπ−2α^i+1,jγ^i+1,jFi+1,jxπ.

Finally, *S*_*i*_′(*x*_*i*+1_, *y*) is positive if *V*_*i*_, *i* = 0,1, 2 …, 11 are positive. This yields the following constraints on the free parameters:

(34) $$\begin{array}{c} {\hat{\beta }}_{i+1,j}>\frac{2{\hat{\alpha }}_{i+1,j}F_{i+1,j}^{y}}{\pi {\hat{\Delta }}_{i+1,j}},\;\;{\hat{\gamma }}_{i+1,j}>\frac{2{\hat{\delta }}_{i+1,j}F_{i+1,j+1}^{y}}{\pi {\hat{\Delta }}_{i+1,j}}. \end{array}$$

The above discussion can be put forward as the following theorem.

**Theorem 3**

The bicubic partially blended rational trigonometric function defined in (4) visualizes monotone data in view of the monotone surface if in each rectangular grid *I* = [*x*_*i*_, *x*_*i*+1_] × [*y*_*j*_, *y*_*j*+1_], free parameters $\beta_{i,j},\;\;\gamma_{i,j},\;\;\beta_{i,j+1},\;\;\gamma_{i,j+1},\;\;{\hat{\beta }}_{i,j},\;\;{\hat{\gamma }}_{i,j},\;\;{\hat{\beta }}_{i+1,j},\;\;{\hat{\gamma }}_{i+1,j}$ satisfy the following constraints:

(35) $$\begin{array}{c} \beta_{i,j}>\frac{2\alpha_{i,j}F_{i,j}^{x}}{\pi \Delta_{i,j}},\;\;\gamma_{i,j}>\frac{2\delta_{i,j}F_{i+1,j}^{x}}{\pi \Delta_{i,j}},\\ \beta_{i,j+1}>\frac{2\alpha_{i,j+1}F_{i,j+1}^{x}}{\pi \Delta_{i,j+1}},\;\;\gamma_{i,j+1}>\frac{2\delta_{i,j+1}F_{i+1,j+1}^{x}}{\pi \Delta_{i,j+1}},\\ {\hat{\beta }}_{i,j}>\frac{2{\hat{\alpha }}_{i,j}F_{i,j}^{y}}{\pi {\hat{\Delta }}_{i,j}},\;\;{\hat{\gamma }}_{i,j}>\frac{2{\hat{\delta }}_{i,j}F_{i,j+1}^{y}}{\pi {\hat{\Delta }}_{i,j}},\\ {\hat{\beta }}_{i+1,j}>\frac{2{\hat{\alpha }}_{i+1,j}F_{i+1,j}^{y}}{\pi {\hat{\Delta }}_{i+1,j}},\;\;{\hat{\gamma }}_{i+1,j}>\frac{2{\hat{\delta }}_{i+1,j}F_{i+1,j+1}^{y}}{\pi {\hat{\Delta }}_{i+1,j}}. \end{array}$$

The above constraints are rearranged as

(36) $$\begin{array}{c} \beta_{i,j}=l_{i,j}+\max\left\{0,\frac{2\alpha_{i,j}F_{i,j}^{x}}{\pi \Delta_{i,j}}\right\},\;l_{i,j}>0,\\ \gamma_{i,j}=m_{i,j}+\max\left\{\frac{2\delta_{i,j}F_{i+1,j}^{x}}{\pi \Delta_{i,j}}\right\},\;m_{i,j}>0,\\ \beta_{i,j+1}=n_{i,j}+\max\left\{\frac{2\alpha_{i,j+1}F_{i,j+1}^{x}}{\pi \Delta_{i,j+1}}\right\},\;n_{i,j}>0,\\ \gamma_{i,j+1}=o_{i,j}+\max\left\{\frac{2\delta_{i,j+1}F_{i+1,j+1}^{x}}{\pi \Delta_{i,j+1}}\right\},\;o_{i,j}>0,\\ {\hat{\beta }}_{i,j}=r_{i,j}+\max\left\{\frac{2{\hat{\alpha }}_{i,j}F_{i,j}^{y}}{\pi {\hat{\Delta }}_{i,j}}\right\},\;r_{i,j}>0,\\ {\hat{\gamma }}_{i,j}=s_{i,j}+\max\left\{\frac{2{\hat{\delta }}_{i,j}F_{i,j+1}^{y}}{\pi {\hat{\Delta }}_{i,j}}\right\},\;s_{i,j}>0,\\ {\hat{\beta }}_{i+1,j}=t_{i,j}+\max\left\{\frac{2{\hat{\alpha }}_{i+1,j}F_{i+1,j}^{y}}{\pi {\hat{\Delta }}_{i+1,j}}\right\},\;t_{i,j}>0,\\ {\hat{\gamma }}_{i+1,j}=u_{i,j}+\max\left\{\frac{2{\hat{\delta }}_{i+1,j}F_{i+1,j+1}^{y}}{\pi {\hat{\Delta }}_{i+1,j}}\right\},\;u_{i,j}>0. \end{array}$$

**Algorithm 4**

*Step 1*. Take a 3D monotone data set {(*x*_*i*_, *y*_*j*_, *F*_*i*,*j*_), *i* = 0,1, 2,…, *n*; *j* = 0,1, 2,…, *m*}.

*Step 2*. Use the Arithmetic Mean Method to estimate the derivatives *F*_*i*,*j*_^*x*^,   *F*_*i*,*j*_^*y*^, *F*_*i*,*j*_^*xy*^ at knots (note: Step 2 is only applicable if data is not provided with derivatives).

*Step 3*. Compute the values of parameters $\beta_{i,j},\;\;\gamma_{i,j},\;\;\beta_{i,j+1},\;\;\gamma_{i,j+1},\;\;{\hat{\beta }}_{i,j},\;\;{\hat{\gamma }}_{i,j}{,\;\;\hat{\beta }}_{i+1,j},{\;\;\hat{\gamma }}_{i+1,j}$ using Theorem 3.

*Step 4*. Substitute the values of variables from Steps 1–3 in rational trigonometric cubic function (4) to visualize monotone surface through monotone data.

## 6. Numerical Example

**Figure 1:**
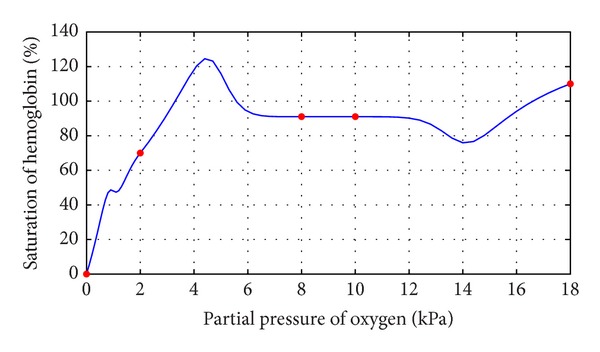
*C* ^1^ rational trigonometric cubic function with *α*_*i*_ = 1.0, *β*_*i*_ = 0.5, *γ*_*i*_ = 1.0,  *δ*_*i*_ = 2.0.

**Figure 2:**
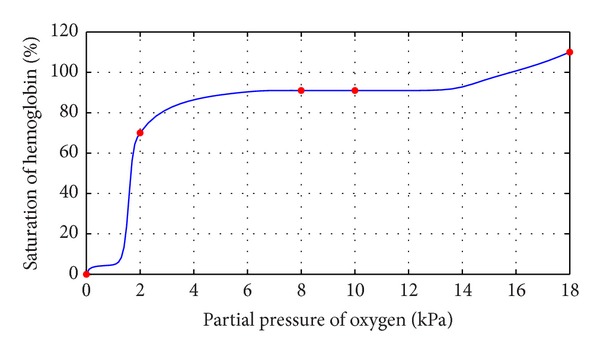
*C* ^1^ monotone rational trigonometric cubic function with *α*_*i*_ = 2.6, *δ*_*i*_ = 0.4.

**Figure 3:**
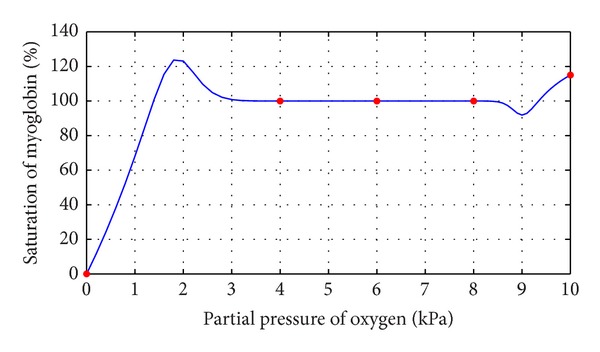
*C* ^1^ rational trigonometric cubic function with *α*_*i*_ = 2.5, *β*_*i*_ = 0.5, *γ*_*i*_ = 0.5,   *δ*_*i*_ = 2.0.

**Figure 4:**
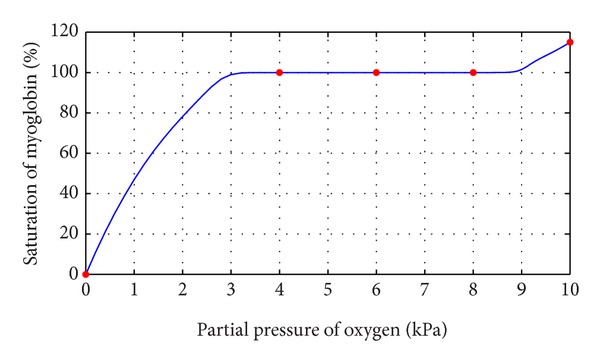
*C* ^1^ monotone rational trigonometric cubic function with *α*_*i*_ = 2.0, *δ*_*i*_ = 0.5.

This section illustrates the monotonicity preserving schemes developed in Sections 4 and 5 with the help of examples. The data in Table 1 is observed by exposing identical samples of hemoglobin to different partial pressures of oxygen which results in varying degree of saturation of hemoglobin with oxygen. The sample obtaining the highest amount is said to be saturated. The amount of oxygen combined with the remaining samples is taken as percentage of this maximum value. At a low partial pressure of oxygen, the percentage saturation of hemoglobin is very low; that is, hemoglobin is combined with only a very little oxygen. At high partial pressure of oxygen, the percentage saturation of hemoglobin is very high; that is, hemoglobin is combined with large amounts of oxygen, that is, a monotone relation, so the resulting curve must exhibit the same behavior.  Figure 1 represents the curve created by assigning random values to free parameters in description of *C* ^1^ rational trigonometric cubic function (1) which does not retain the monotone nature of the data. This impediment is removed by applying monotonicity preserving schemes developed in Section 4 and is shown in Figure 2. It is evident from the figure that this curve preserves the monotone shape of hemoglobin dissociation curve. Similar investigation in Table 2 displays a series of results for percentage saturation of myoglobin and partial pressure of oxygen.  Figure 3 is produced by assigning random values to free parameters in description of *C* ^1^ rational trigonometric cubic function (1) which fails to conserve the monotone trend of data.  Algorithm 2 developed in Section 4 is applied to remove this drawback and Figure 4 displays the required result. Numerical results corresponding to Figures 2 and 4 are shown in Tables 3 and 4.

The 3D monotone data set in Tables 5 and 6 are generated from the following functions:

(37) $$\begin{array}{c} F\left(x,y\right)=\sqrt{\frac{x^{2}}{25}+\frac{y^{2}}{16}},\\ F\left(x,y\right)=\log\left(x^{2}+y^{2}\right). \end{array}$$

respectively.

**Figure 5:**
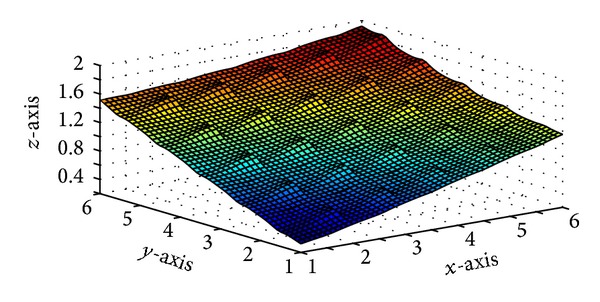
*C* ^1^ rational trigonometric bicubic function with *α*_*i*,*j*_ = 14, *β*_*i*,*j*_ = 15, *γ*_*i*,*j*_ = 6,  *δ*_*i*,*j*_ = 7,  *α*_*i*,*j*+1_ = 8,  *β*_*i*,*j*+1_ = 8,  *γ*_*i*,*j*+1_ = 4, *δ*_*i*,*j*+1_ = 9,  ${\hat{\alpha }}_{i,j}=6$, ${\hat{\beta }}_{i,j}=5$,  ${\hat{\gamma }}_{i,j}=4$,  ${\hat{\delta }}_{i,j}=8$, ${\hat{\alpha }}_{i+1,j}=15$,  ${\hat{\beta }}_{i+1,j}=2$,  ${\hat{\gamma }}_{i+1,j}=12$,  ${\hat{\delta }}_{i+1,j}=8$.

**Figure 6:**
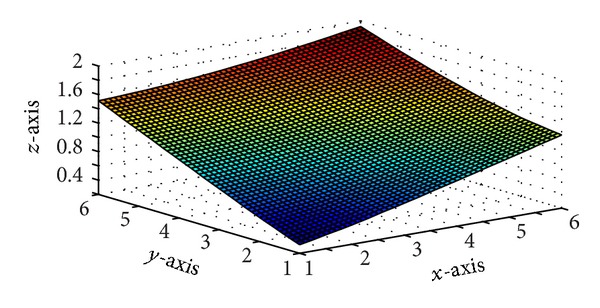
*C* ^1^ monotone rational trigonometric bicubic function with *α*_*i*,*j*_ = 12,  *δ*_*i*,*j*_ = 12, *α*_*i*,*j*+1_ = 14,  *δ*_*i*,*j*+1_ = 13,  ${\hat{\alpha }}_{i,j}=12$,  ${\hat{\delta }}_{i,j}=12$,  ${\hat{\alpha }}_{i+1,j}=15$, ${\hat{\delta }}_{i+1,j}=13$.

**Figure 7:**
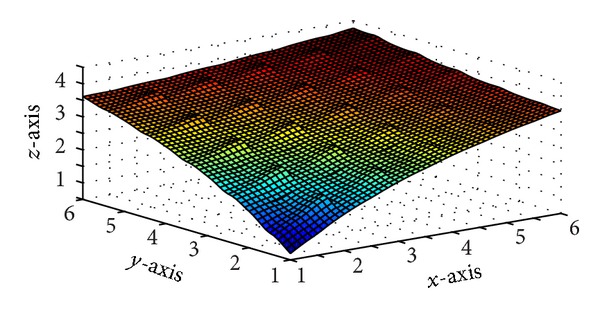
*C* ^1^ rational trigonometric bicubic function with *α*_*i*,*j*_ = 14,  *β*_*i*,*j*_ = 8,  *γ*_*i*,*j*_ = 5, *δ*_*i*,*j*_ = 7,  *α*_*i*,*j*+1_ = 14,  *β*_*i*,*j*+1_ = 8,  *γ*_*i*,*j*+1_ = 6,  *δ*_*i*,*j*+1_ = 13,  ${\hat{\alpha }}_{i,j}=12$, ${\hat{\beta }}_{i,j}=9$,  ${\hat{\gamma }}_{i,j}=8$,  ${\hat{\delta }}_{i,j}=8$,  ${\hat{\alpha }}_{i+1,j}=15$,  ${\hat{\beta }}_{i+1,j}=9$,  ${\hat{\gamma }}_{i+1,j}=8$, ${\hat{\delta }}_{i+1,j}=8$.

**Figure 8:**
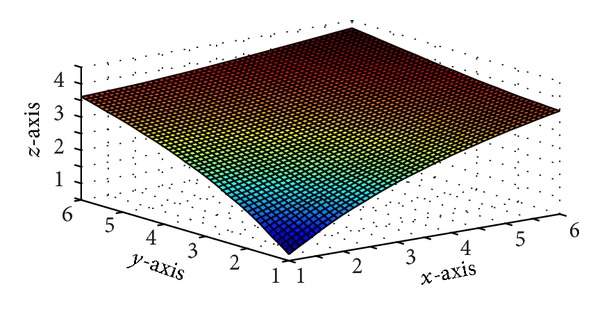
*C* ^1^ monotone rational trigonometric bicubic function with *α*_*i*,*j*_ = 12,  *δ*_*i*,*j*_ = 12,  *α*_*i*,*j*+1_ = 13,  *δ*_*i*,*j*+1_ = 14,  ${\hat{\alpha }}_{i,j}=10$,  ${\hat{\delta }}_{i,j}=10$,  ${\hat{\alpha }}_{i+1,j}=15$,  ${\hat{\delta }}_{i+1,j}=13$.

Figures 5 and 7 are produced by interpolating the monotone data sets in Tables 5 and 6, respectively, by *C* ^1^ rational trigonometric bicubic function for arbitrary values of free parameter. Monotone surfaces in Figures 6 and 8 are produced by interpolating the same data by the monotonicity preserving scheme developed in Section 5. Tables 7 and 8 enclose numerical results against Figures 6 and 8.

## 7. Conclusion 

In this paper, monotonicity of data is retained by developing constraints on free parameters in the specification of rational trigonometric function and bicubic blended function. Authors in [7, 8] used algebraic function while the proposed algorithm applies trigonometric function which gives much smoother result due to orthogonality of sine and cosine function. Shape preserving techniques of Butt and Brodlie [1] required insertion of additional knots. In [12], developed scheme failed to maintain smoothness. The proposed technique is local, affirms smoothness, works well for data with derivatives, and does not require insertion of extra knots. Derivative of trigonometric spline is much lower than that of polynomial spline.

## Figures and Tables

**Table 1:** The varying ability of hemoglobin to carry oxygen.

Partial pressure of oxygen (kPa)	0	2	8	10	18
Saturation of hemoglobin (%)	0	70	91	91	110

**Table 2:** The varying ability of myoglobin.

Partial pressure of oxygen (kPa)	0	4	6	8	10
Saturation of myoglobin (%)	0	100	100	100	115

**Table 3:** Numerical results corresponding to Figure 2.

*i*	1	2	3	4	5
*d*_*i*_	50.9065	19.6819	0	0	5.7983
*β*_*i*_	35.01	7.17	0	0.01	—
*γ*_*i*_	0.1890	0.0100	0	0.7871	—

**Table 4:** Numerical results corresponding to Figure 4.

*i*	1	2	3	4	5
*d*_*i*_	56.25	0	0	0	15
*β*_*i*_	2.8748	9.3179	0	0.01	—
*γ*_*i*_	0.01	0.01	0	0.6466	—

**Table 5:** A 3D monotone data set.

*y*/*x*	1	2	3	4	5	6
1	0.3202	0.5385	0.7762	1.0198	1.2659	1.5133
2	0.4717	0.6403	0.8500	1.0770	1.3124	1.5524
3	0.6500	0.7810	0.9605	1.1662	1.3865	1.6155
4	0.8382	0.9434	1.0966	1.2806	1.4841	1.7000
5	1.0308	1.1180	1.2500	1.4142	1.6008	1.8028
6	1.2258	1.3000	1.4151	1.5620	1.7328	1.9209

**Table 6:** A 3D monotone data set.

*y*/*x*	1	2	3	4	5	6
1	0.6931	1.6094	2.3026	2.8332	3.2581	3.6109
2	1.6094	2.0794	2.5649	2.9957	3.3673	3.6889
3	2.3026	2.5649	2.8904	3.2189	3.5264	3.8067
4	2.8332	2.9957	3.2189	3.4657	3.7136	3.9512
5	3.2581	3.3673	3.5264	3.7136	3.9120	4.1109
6	3.6109	3.6889	3.8067	3.9512	4.1109	4.2767

**Table 7:** Numerical values corresponding to Figure 6.

(*x*_*i*_, *y*_*j*_)	1	2	3	4	5	6
Numerical values of *F*_*i*,*j*_^*x*^						
1	0.1382	0.0823	0.0555	0.0413	0.0328	0.0271
2	0.1649	0.1213	0.0921	0.0732	0.0603	0.0511
3	0.1832	0.1515	0.1233	0.1018	0.0858	0.0738
4	0.1904	0.1685	0.1448	0.1240	0.1071	0.0936
5	0.1938	0.1783	0.1593	0.1407	0.1243	0.1105
6	0.1962	0.1856	0.1709	0.1550	0.1396	0.1259

Numerical values of *F*_*i*,*j*_^*y*^						
1	0.2087	0.2280	0.2406	0.2448	0.2467	0.2480
2	0.1481	0.1892	0.2184	0.2312	0.2377	0.2423
3	0.1068	0.1552	0.1926	0.2130	0.2247	0.2333
4	0.0813	0.1292	0.1686	0.1937	0.2097	0.2221
5	0.0649	0.1096	0.1481	0.1754	0.1943	0.2097
6	0.0538	0.0947	0.1310	0.1588	0.1794	0.1969

Numerical values of *β*_*i*,*j*_						
1	10.9406	9.7062	9.0177	8.6526	8.4470	—
2	11.0996	10.3406	10.0079	9.8513	9.7684	—
3	11.6858	11.1996	10.8694	10.6747	10.5583	—
4	11.8607	11.5787	11.3235	11.1397	11.0149	—
5	11.9272	11.7583	11.5754	11.4218	11.3048	—
6	—	—	—	—	—	—

Numerical values of *γ*_*i*,*j*_						
1	13.0594	14.2938	14.9823	15.3474	15.5530	—
2	12.3315	12.9236	13.3931	13.7011	13.8977	—
3	12.1426	12.4531	12.7625	13.0043	13.1785	—
4	12.0737	12.2518	12.4569	12.6399	12.7863	—
5	12.0728	12.2417	12.4246	12.5782	12.6952	—
6	—	—	—	—	—	—

Numerical values of ${\hat{\beta }}_{i,j}$						
1	11.4688	11.5120	11.8546	11.9391	11.9689	—
2	10.5384	10.8247	11.5416	11.7866	11.8858	—
3	9.7828	10.3810	11.2336	11.6016	11.7732	—
4	9.2668	10.1222	10.9942	11.4274	11.6537	—
5	8.9258	9.9673	10.8217	11.2811	11.5418	—
6	—	—	—	—	—	—

Numerical values of ${\hat{\gamma }}_{i,j}$						
1	12.5312	12.1490	12.0616	12.0312	12.0311	—
2	13.4616	12.4963	12.2213	12.1165	12.1142	—
3	14.2172	12.8787	12.4267	12.2357	12.2268	—
4	14.7332	13.2084	12.6331	12.3675	12.3463	—
5	15.0742	13.4662	12.8168	12.4961	12.4582	—
6	—	—	—	—	—	—

Numerical values of *β*_*i*,*j*+1_						
1	11.3239	10.5207	10.0947	9.8548	9.7100	—
2	12.0640	11.6759	11.4932	11.3965	11.3401	—
3	13.0662	12.6810	12.4538	12.3180	12.2329	—
4	13.5085	13.2108	12.9963	12.8508	12.7519	—
5	13.7180	13.5047	13.3254	13.1890	13.0885	—
6	—	—	—	—	—	—

Numerical values of *γ*_*i*,*j*+1_						
1	15.4850	16.2308	16.6264	16.8491	16.9836	—
2	14.0006	14.5092	14.8428	15.0559	15.1949	—
3	13.4909	13.8260	14.0880	14.2768	14.4104	—
4	13.2728	13.4950	13.6932	13.8518	13.9731	—
5	13.2618	13.4600	13.6264	13.7531	13.8464	—
6	—	—	—	—	—	—

Numerical values of ${\hat{\beta }}_{i+1,j}$						
1	9.4938	11.1409	12.5497	13.1538	13.4520	—
2	8.8689	10.3644	11.8756	12.6696	13.1076	—
3	8.6842	10.0813	11.4747	12.3097	12.8189	—
4	8.6336	10.0176	11.2653	12.0687	12.5971	—
5	8.6325	10.0429	11.1705	11.9192	12.4370	—
6	—	—	—	—	—	—

Numerical values of ${\hat{\gamma }}_{i+1,j}$						
1	14.5834	13.5377	13.2398	13.1262	13.1238	—
2	15.4020	13.9519	13.4623	13.2553	13.2457	—
3	15.9609	14.3091	13.6858	13.3981	13.3751	—
4	16.3304	14.5884	13.8848	13.5375	13.4964	—
5	16.5779	14.7990	14.0514	13.6641	13.6025	—
6	—	—	—	—	—	—

**Table 8:** Numerical values corresponding to Figure 8.

(*x*_*i*_, *y*_*j*_)	1	2	3	4	5	6
Numerical values of *F*_*i*,*j*_^*x*^						
1	1.0279	0.4623	0.2308	0.1322	0.0843	0.0581
2	0.8047	0.4778	0.2939	0.1928	0.1341	0.0979
3	0.6119	0.4581	0.3270	0.2350	0.1731	0.1312
4	0.4778	0.4012	0.3180	0.2473	0.1928	0.1521
5	0.3889	0.3466	0.2939	0.2428	0.1987	0.1627
6	0.3168	0.2966	0.2667	0.2326	0.1991	0.1689

Numerical values of *F*_*i*,*j*_^*y*^						
1	1.0279	0.8047	0.6119	0.4778	0.3889	0.3168
2	0.4623	0.4778	0.4581	0.4012	0.3466	0.2966
3	0.2308	0.2939	0.3270	0.3180	0.2939	0.2667
4	0.1322	0.1928	0.2350	0.2473	0.2428	0.2326
5	0.0843	0.1341	0.1731	0.1928	0.1987	0.1991
6	0.0581	0.0979	0.1312	0.1521	0.1627	0.1689

Numerical values of *β*_*i*,*j*_						
1	13.4612	11.8021	10.5579	9.7618	9.2601	—
2	13.9316	11.8084	10.8374	10.3699	10.1191	—
3	13.8377	12.7622	11.9437	11.4236	11.0979	—
4	13.4933	12.9563	12.4102	11.9764	11.6602	—
5	13.2255	12.9325	12.5819	12.2566	11.9879	—
6	—	—	—	—	—	—

Numerical values of *γ*_*i*,*j*_						
1	10.5388	12.1979	13.4421	14.2382	14.7399	—
2	10.5932	11.3237	12.0568	12.6377	13.0617	—
3	10.8043	11.1752	11.6161	12.0237	12.3602	—
4	10.9824	11.1929	11.4696	11.7539	12.0121	—
5	10.7745	11.0675	11.4181	11.7434	12.0121	—
6	—	—	—	—	—	—

Numerical values of ${\hat{\beta }}_{i,j}$						
1	13.4612	13.9316	13.8377	13.4933	13.2255	—
2	11.8021	11.8084	12.7622	12.9563	12.9325	—
3	10.5579	10.8374	11.9437	12.4102	12.5819	—
4	9.7618	10.3699	11.4236	11.9764	12.2566	—
5	9.2601	10.1191	11.0979	11.6602	11.9879	—
6	—	—	—	—	—	—

Numerical values of ${\hat{\gamma }}_{i,j}$						
1	10.5388	10.5932	10.8043	10.9824	10.7745	—
2	12.1979	11.3237	11.1752	11.1929	11.0675	—
3	13.4421	12.0568	11.6161	11.4696	11.4181	—
4	14.2382	12.6377	12.0237	11.7539	11.7434	—
5	14.7399	13.0617	12.3602	12.0121	12.0121	—
6	—	—	—	—	—	—

Numerical values of *β*_*i*,*j*+1_						
1	13.7691	12.3176	11.3888	10.8035	10.4245	—
2	13.7765	12.6436	12.0982	11.8056	11.6334	—
3	14.8893	13.9343	13.3275	12.9476	12.7025	—
4	15.1157	14.4785	13.9724	13.6036	13.3401	—
5	15.0879	14.6788	14.2994	13.9859	13.7398	—
6	—	—	—	—	—	—

Numerical values of *γ*_*i*,*j*+1_						
1	13.2144	14.5622	15.4247	15.9682	16.3201	—
2	12.2673	13.0616	13.6908	14.1502	14.4789	—
3	12.1065	12.5841	13.0257	13.3902	13.6766	—
4	12.1257	12.4254	12.7334	13.0131	13.2509	—
5	11.9898	12.3697	12.7220	13.0131	13.2416	—
6	—	—	—	—	—	—

Numerical values of ${\hat{\beta }}_{i+1,j}$						
1	7.0627	9.6496	12.0876	13.2188	13.7521	—
2	6.8759	8.4746	10.6260	11.9816	12.7945	—
3	7.0547	8.2958	10.0152	11.2619	12.1246	—
4	7.2590	8.4154	9.8191	10.8928	11.7015	—
5	7.4425	8.6142	9.8100	10.7305	11.4556	—
6	—	—	—	—	—	—

Numerical values of ${\hat{\gamma }}_{i+1,j}$						
1	13.2144	12.2673	12.1065	12.1257	11.9898	—
2	14.5622	13.0616	12.5841	12.4254	12.3697	—
3	15.4247	13.6908	13.0257	12.7334	12.7220	—
4	15.9682	14.1502	13.3902	13.0131	13.0131	—
5	16.3201	14.4789	13.6766	13.2509	13.2416	—
6	—	—	—	—	—	—
